# Quality for All: Clinical Trial Enrollment and End‐of‐Life Care in Solid and Hematologic Malignancies

**DOI:** 10.1002/cam4.70775

**Published:** 2025-04-27

**Authors:** Melissa R. Rosen, Tracy Truong, Catherine Gervais, Thomas W. LeBlanc, Laura J. Havrilesky, Brittany A. Davidson

**Affiliations:** ^1^ Department of Gynecology and Obstetrics Johns Hopkins University Baltimore Maryland USA; ^2^ Department of Biostatistics and Bioinformatics Duke University School of Medicine Durham North Carolina USA; ^3^ Department of Hematologic Malignancies Duke University Medical Center Durham North Carolina USA; ^4^ Department of Obstetrics and Gynecology Duke University Medical Center Durham North Carolina USA

**Keywords:** clinical trials, end‐of‐life care, goals of care

## Abstract

**Background:**

Patients with incurable cancer deserve quality end‐of‐life (EOL) care. Despite established EOL quality metrics, many patients receive aggressive EOL care with limited goals of care (GOC) documentation. Concurrently, clinical trials are critical for advancing cancer care. We aim to identify associations between trial enrollment in the last year of life (YOL) and EOL quality metrics for adults with cancer to identify opportunities to advance goal‐concordant care.

**Methods:**

This is a retrospective review of adult patients with cancer at a single academic institution who died between January 2018 and October 2022. Outcomes included: initiation of a new anticancer therapy, intensive care unit (ICU) admission, hospitalization, or emergency department (ED) encounter in the last 30 days of life (DOL), reception of anti‐cancer treatment in the last 14 DOL, referral to hospice, referral to palliative care, and GOC documentation.

**Results:**

Among 9817 patients, 577 (5.9%) enrolled in clinical trials in the last YOL. Patients enrolled in trials were more likely to initiate new anticancer treatments in the last 30 DOL (*p* = < 0.001), less likely to have a palliative care referral (*p* = < 0.001) or GOC documentation (*p* = < 0.001), but were less likely to have an ED encounter in the last 30 DOL (*p* = 0.04) or die in an acute care setting (*p* = 0.015).

**Conclusions:**

Enrollment in clinical trials in the last YOL was associated with metrics of aggressive EOL care, with low rates of GOC documentation to determine if this care is goal‐concordant. Low rates of palliative care and hospice engagement across the study population suggest opportunities for improvement for all patients, regardless of trial enrollment.

## 1 Introduction

Ensuring high‐quality cancer care is essential throughout the cancer continuum, from diagnosis to end‐of‐life (EOL) care. Too often, however, patients with advanced or recurrent malignancies receive interventions or anticancer treatments that do not align with their goals of care. (GOC) [1, 2, 3, 4]. The Institute of Medicine's reports “Crossing the Quality Chasm” and “Ensuring Quality Cancer Care,” emphasized the importance of adopting a patient‐centered approach during clinical encounters, empowering patients to play an active role in their healthcare [5, 6]. This approach has been supported by national organizations such as the National Quality Forum (NQF) and the American Society of Clinical Oncology (ASCO), which have developed evidence‐based guidelines to improve the delivery of goal‐concordant, quality cancer care near the EOL, in an effort to limit aggressive, ineffective care. Specifically, these organizations discourage the initiation of new anticancer regimens in the last month of life, reception of anticancer treatment in the last 2 weeks of life, and enrollment in hospice services in the last 3 days of life (DOL) [2, 7]. Furthermore, these recommendations encourage early and frequent GOC discussions to ensure that treatment recommendations align with patient‐reported values alongside early integration of specialist palliative care (PC) [2, 7]. Ample data demonstrate that these key interventions improve patient and caregiver quality of life and limit aggressive EOL care without shortening survival (and possibly even prolonging it) [8, 9, 10, 11]. Despite established guidelines, these supports are not broadly utilized in day‐to‐day practice and EOL care remains an area that is often neglected until late in the disease course, if at all [1, 2, 9, 12, 13].

As patients evaluate their options for cancer‐directed therapy, many will choose to enroll in a clinical trial, gaining access to novel treatments that may evolve the standard of care and offer survival or quality benefits. At the 64 National Cancer Institute (NCI)‐designated cancer centers, 19% of patients will participate in a clinical trial over the course of their cancer trajectory [14, 15]. Trial participation is vital for the development of innovative anticancer therapies and the advancement of medical practice. It is crucial that involvement in clinical trials aligns with patients' goals and meets standard measures of high‐quality care, especially when patients enroll in trials near the end of their lives. Previous studies investigating the EOL experience of patients enrolled in clinical trials have shown trends toward the reception of more aggressive EOL care. However, these studies were limited by small sample sizes and narrow scopes of inquiry [16, 17, 18].

The aim of this study is to evaluate the NQF's EOL quality metrics and clinical trial participation within 12 months of death, identifying potential gaps in EOL care for patients that can improve the quality of care that patients with cancer receive.

## 2 Methods

This is a retrospective cohort study of adult patients diagnosed with solid or hematologic malignancies who died of cancer between January 2018 and October 2022. All study activities occurred following Duke University Institutional Review Board approval (Pro00110974). All subjects were treated at a single NCI‐designated comprehensive cancer center comprised of three hospitals and were identified using an institutional tumor registry. Eligible patients included those ≥ 18 years of age at death with active oncologic disease at the time of death seen for care at one of the three Duke University‐affiliated hospitals. Active oncologic disease was defined by the documentation of an oncology treatment plan in the electronic health record (EHR) in the last 12 months of life, receipt of an oral or intravenous anticancer agent in the last 12 months of life, or the completion of > 3 outpatient oncology appointments at one of the three Duke University Hospitals in the last year of life, as defined by the authors previously [19]. To identify patients eligible for inclusion by number of clinic visits in a 12‐month period, two co‐authors reviewed objective clinic attendance records in the EHR.

Clinical and demographic data for study subjects were abstracted from the EHR, including date of birth, date of death, sex, marital status, patient‐reported race, primary cancer site, date of diagnosis, and clinical trial participation. For subjects enrolled in a clinical trial, the date of trial enrollment and trial phase were recorded. Subjects were considered part of the clinical trial cohort if they were enrolled in a pharmacologic clinical trial in the last 12 months of life. Those with incomplete data were excluded from the analysis.

The primary outcomes of this study were the EOL quality metrics, as defined by established NQF‐endorsed measures, including initiation of a new anticancer regimen in the last 30 DOL, reception of any anticancer therapy in the last 14 DOL, and time from hospice referral to death of < 3 days [7]. Secondary outcomes included timing of hospice referral and specialist PC involvement, documentation of GOC, healthcare power of attorney (HCPOA) and advance directives in the EHR, hospitalization, admission to the intensive care unit (ICU), or emergency department (ED) encounter in the last 30 DOL, and location of death. GOC documentation was defined as the presence of an advance care planning note in the EHR. These could have been written by a variety of healthcare providers, including oncologists and palliative care physicians and advance practice providers.

Patient demographics and outcomes were summarized using descriptive statistics. Continuous variables were described using the mean and standard deviation (SD) or the median and interquartile range and were compared using two‐sample *t*‐tests or Wilcoxon rank‐sum tests. Categorical variables were described with frequencies and percentages and compared using chi‐square or Fisher's exact tests. Quality of care measures were compared by clinical trial enrollment status. All statistical analyses were performed in R 4.2.2 (R Core Team, 2022) at a 0.05 two‐tailed level of significance.

## 3 Results

### 3.1 Cohort Demographics

**FIGURE 1:**
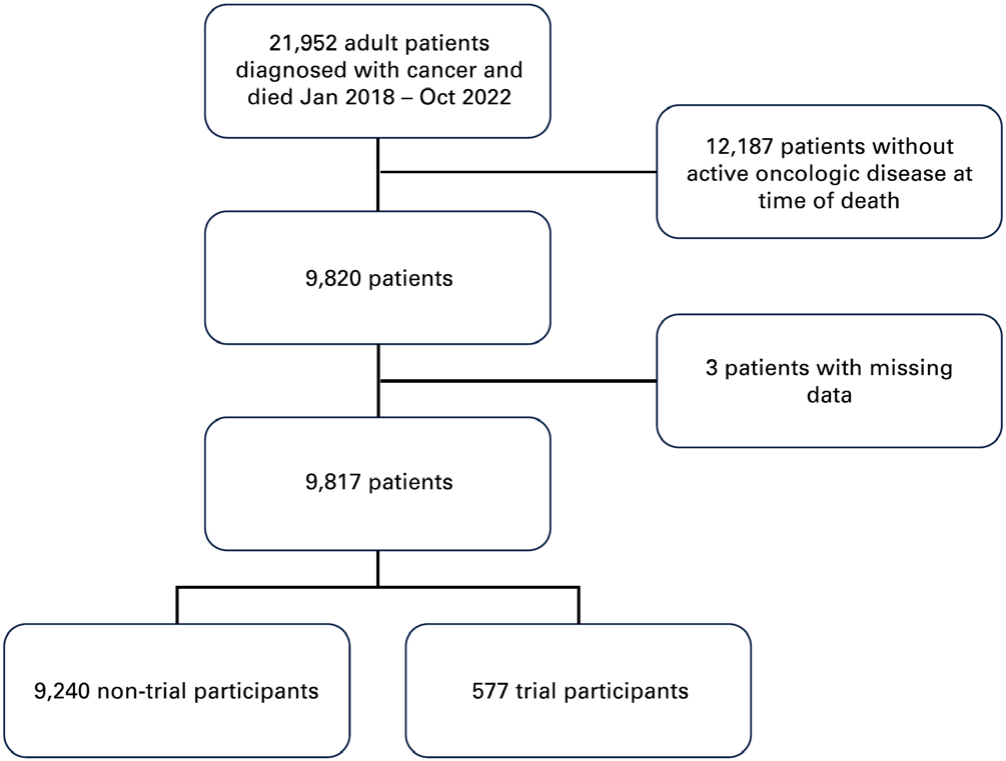
Flowchart of patient cohort.

We identified 21,952 patients with cancer who received care at our center and died between January 2018 and October 2022. Of these, 9820 met eligibility criteria. Three patients with incomplete medical records were excluded from the analysis. A total of 9817 patients were included in the final cohort (Figure 1). Patient demographics are summarized in Table 1. Five hundred and seventy‐seven patients (5.9%) were enrolled in at least one clinical trial in the last 12 months of life, with more than half (51.3%; 296/577) enrolling within the last 6 months of life. One hundred and forty‐six patients (25.3%) participated in a phase I trial, 82 (14.2%) in phase I/II, 254 (44.0%) in phase II, 16 (2.8%) in phase II/III, 60 (10.4%) in phase III, 16 (2.8%) in phase IV, and 3 (0.5%) in an expanded access trial. The most common primary cancer sites in the trial group were neurologic, gastrointestinal, and thoracic, while in the nontrial group were gastrointestinal, thoracic, and genitourinary. About 58.4% of the trial group and 53.8% of the nontrial group were male. Among trial subjects, 83.4% self‐identified as White and 14.2% as Black, while 69.9% of nontrial subjects were White and 23.8% were Black. Trial subjects were 14% more likely to be married or in a partnership than nontrial subjects.

**TABLE 1:** Characteristics of patients with cancer diagnoses who died between January 2018—October 2022.

Characteristics	Nontrial subjects, *N* (%) (*n* = 9240)	Trial subjects, *N* (%) (*n* = 577)	Total *N* (%) (*n* = 9817)
Age at diagnosis			
Mean (SD)	64.4 (13.2)	58.8 (12.4)	64.1 (13.2)
Median (IQR)	66 [57, 73]	60 [52, 68]	65 [57, 73]
Age at death			
Mean (SD)	67.6 (12.8)	62 (12.3)	67.2 (12.8)
Median (IQR)	69 [60, 76]	64 [56, 71]	69 [60, 76]
Sex			
Female	4266 (46.2%)	240 (41.6%)	4506 (45.9%)
Male	4971 (53.8%)	337 (58.4%)	5308 (54.1%)
Other	3 (0.0%)	0 (0.0%)	3 (0.0%)
Race/ethnicity			
American Indian or Alaskan Native	60 (0.6%)	1 (0.2%)	61 (0.6%)
Asian	128 (1.4%)	2 (0.3%)	130 (1.3%)
Black or African American, non‐Hispanic	2199 (23.8%)	82 (14.2%)	2281 (23.2%)
Hispanic	138 (1.5%)	3 (0.5%)	141 (1.4%)
Native Hawaiian or Pacific Islander	9 (0.1%)	0 (0.0%)	9 (0.1%)
Other/unknown	248 (2.7%)	11 (1.9%)	259 (2.6%)
White, non‐Hispanic	6458 (69.9%)	478 (82.8%)	6936 (70.7%)
Marital status			
Single/divorced/separated/widowed	3591 (38.9%)	147 (25.5%)	3738 (38.1%)
Married/living with partner	5464 (60.3%)	419 (74%)	5883 (61.1%)
Unknown	185 (2.0%)	11 (1.9%)	196 (2.0%)
Primary tumor site			
Breast	643 (7.0%)	27 (4.7%)	670 (6.8%)
Dermatologic	166 (1.8%)	10 (1.7%)	176 (1.8%)
Endocrine	50 (0.5%)	0 (0.0%)	50 (0.5%)
Gastrointestinal	2175 (23.5%)	109 (18.9%)	2284 (23.3%)
Genitourinary	1054 (11.4%)	60 (10.4%)	1114 (11.3%)
Gynecologic	555 (6.0%)	31 (5.4%)	586 (6.0%)
Head & neck	228 (2.5%)	13 (2.3%)	241 (2.5%)
Leukemia	956 (10.3%)	80 (13.9%)	1036 (10.6%)
Lymphoma	391 (4.2%)	16 (2.8%)	407 (4.1%)
Musculoskeletal	167 (1.8%)	12 (2.1%)	179 (1.8%)
Neurologic	777 (8.4%)	121 (21.0%)	898 (9.2%)
Ophthalmologic	28 (0.3%)	4 (0.7%)	32 (0.3%)
Thoracic	1887 (20.4%)	90 (15.6%)	1977 (20.1%)
Unknown	163 (1.8%)	4 (0.7%)	167 (1.7%)

Abbreviations: IQR, interquartile range; SD, standard deviation.

### 3.2 EOL Quality Metrics

EOL quality measures were evaluated by clinical trial enrollment status across the study cohort (Table 2). In the last month of life, 19.4% of clinical trial subjects initiated a new cancer‐directed therapy compared to 14.2% of nontrial subjects (*p* < 0.001). However, subjects enrolled in trials at the EOL were just as likely to receive anticancer treatment within 14 days of death (7.8% vs. 5.8%; *p* = 0.055). There were no differences in referrals to hospice in the last three DOL, with 11 trial subjects (8.3%) and 206 (9.1%) nontrial subjects referred during this timeframe (*p* = 0.88). Trial enrollment was associated with a lower likelihood of specialist PC referral (21.8% vs. 28.1% of nontrial subjects; *p* = < 0.001). Both trial and nontrial subjects were referred to PC services relatively late in their cancer trajectory (median of 41 vs. 42 days before death, respectively; *p* = 0.59). Similarly, a low proportion of hospice referrals were seen in both groups and did not differ by study cohort (22.9% of trial vs. 24.5% of nontrial subjects referred; *p* = 0.42). Furthermore, there was no difference in the median days from hospice referral to death (19 vs. 22 days for the trial and nontrial groups, respectively; *p* = 0.22).

**TABLE 2:** End‐of‐life quality metrics of patients with cancer diagnoses who died between January 2018 and October 2022.

Characteristics	Nontrial subjects, *N* (%) (*n* = 9240)	Trial subjects, *N* (%) (*n* = 577)	Total *N* (%) (*n* = 9817)	*p*
Years from cancer diagnosis to death				
Median [IQR]	1.5 [0.6, 3.7]	1.8 [1, 3.8]	1.5 [0.6, 3.7]	< 0.0001*
Min, max	0, 58.1	0.1, 26.7	0, 58.1	
*N* observed	9182	575	9757	
Missing	58 (0.63%)	2 (0.35%)	60 (0.61%)	
Receipt of anticancer therapy in last 14 DOL	534 (5.8%)	45 (7.8%)	579 (5.9%)	0.055
Initiation of new anticancer regimen in last 30 DOL	1308 (14.2%)	112 (19.4%)	1420 (14.5%)	0.0008*
ICU admission in last 30 DOL	121 (1.3%)	7 (1.2%)	128 (1.3%)	1
Number of ICU admissions				
0	9119 (98.7%)	570 (98.8%)	9689 (98.7%)	0.91
1	111 (1.2%)	7 (1.2%)	118 (1.2%)	
2+	10 (0.1%)	0 (0.0%)	10 (0.1%)	
ED encounter in last 30 DOL	2823 (30.6%)	153 (26.5%)	2976 (30.3%)	0.045*
Number of ED encounters				
0	6417 (69.4%)	424 (73.5%)	6841 (69.7%)	0.1
1	2152 (23.3%)	113 (19.6%)	2265 (23.1%)	
2+	671 (7.3%)	40 (6.9%)	711 (7.3%)	
Hospital admission in last 30 DOL	3338 (36.1%)	198 (34.3%)	3536 (36.0%)	0.4
Number of hospital admissions				
0	5902 (63.9%)	379 (65.7%)	6281 (64.0%)	0.69
1	2542 (27.5%)	150 (26.0%)	2692 (27.4%)	
2+	796 (8.6%)	48 (8.3%)	844 (8.6%)	
Referred to palliative care	2597 (28.1%)	126 (21.8%)	2723 (27.7%)	0.0009*
Time from palliative care referral to date of death, median days [IQR]	42 [12, 138]	41 [15, 147]	42 [13, 138]	0.59
Referred to hospice	2261 (24.5%)	132 (22.9%)	2393 (24.4%)	0.42
Hospice referral to death < 3 days duration	206 (9.1%)	11 (8.3%)	217 (9%)	0.88
Time from hospice referral to date of death, median days [IQR]	22 [9, 50]	19 [7.8, 42.5]	21 [9, 50]	0.22
Death in an acute care setting	1540 (16.7%)	74 (12.8%)	1614 (16.4%)	0.015*
HCPOA document uploaded to EHR	1680 (18.2%)	109 (18.9%)	1789 (18.2%)	0.66
Advanced directive uploaded to EHR	1384 (15%)	89 (15.4%)	1473 (15%)	0.76
GOC note uploaded to EHR	2362 (25.6%)	107 (18.5%)	2469 (25.2%)	0.0001*

Abbreviations: DOL, days of life; ED, emergency department; EHR, electronic health record; GOC, goals of care; HCPOA, healthcare power of attorney; ICU, intensive care unit; IQR, interquartile range.

* Statistically significant.

GOC documentation in the EHR was higher among nontrial subjects compared to trial subjects (25.6% vs. 18.5%; *p* < 0.001). Advance directive documentation was infrequent, with 15% of trial and nontrial subjects having an advance directive document entered into the EHR (*p* = 0.76). There was similarly infrequent HCPOA documentation in the EHR (18.9% of trial and 18.2% of nontrial subjects, *p* = 0.66).

More than 1 in 4 (26.5%) trial subjects had an ED encounter in the last month of life, though this was higher among nontrial subjects (30.6%; *p* = 0.04). Furthermore, 6.9% of trial‐enrolled participants sought care in the ED on 2 or more occasions, compared to 7.3% of nontrial participants. More than one‐third of patients were hospitalized during their last month of life (34.3% trial vs. 36.1% nontrial, *p* = 0.69) though ICU admissions were low (1.2%) for both groups. 8.3% of trial subjects and 8.6% of nontrial subjects were hospitalized more than once during their last month of life. Clinical trial subjects had a lower likelihood of dying in an acute care setting (12.8% vs. 16.7%; *p* = 0.015).

## 4 Discussion

Our study was an evaluation of EOL quality of care metrics in nearly 10,000 patients with cancer in the last year of life to understand how these differed by clinical trial enrollment in the same time period. We identified increased rates of new anticancer regimens in the last 30 DOL among patients who had enrolled in clinical trials at some point in their last year of life. These rates of seemingly high‐intensity EOL care may be warranted; however, if they align with patients' values and GOC, following the pursuit of goal‐concordant cancer care delivery, as recommended by ASCO [20]. The challenge in this is overall low rates of GOC documentation; in our study, only 25.2% of patients in the overall cohort had documented GOC conversations in their EHR, making it nearly impossible to ascertain if this aggressive EOL care was consistent with patients' own care preferences. An appropriately timed shift from cancer‐directed therapy to a focus on comfort and symptom control is essential for both patients and caregivers, as adverse side effects and treatment‐related time toxicity may conflict with patients' EOL goals [2, 21].

Our study demonstrates that nearly 20% of patients in the clinical trial cohort started a new cancer‐directed treatment in their last month of life. Previous studies of EOL care in clinical trial subjects have yielded similar trends, suggesting that trial subjects are at an increased risk of receiving anticancer treatment near the EOL [16, 17]. Thompson et al. report that trial subjects were three times more likely to receive chemotherapy in the last 2 months of life, and Nitecki et al. report that trial subjects with ovarian cancer were more likely to start a new anticancer treatment in the last month of life; however, neither report on rates of GOC discussions or PC involvement, which may help in facilitating some of these conversations [16, 17].

Although we cannot establish that the desire to participate in a clinical trial in the last year of life is the causative factor in the increased incidence of aggressive EOL care among these patients, it is important to acknowledge that this group may be more likely to receive care that is widely regarded as overly aggressive. Directed quality improvement efforts toward increased rates of comprehensive, longitudinal GOC discussions (and the documentation of these conversations) are warranted to ensure that the care patients receive is truly in line with their values and healthcare goals. Cancer‐directed treatment should be individualized, and it is our duty as oncologic care providers to effectively engage patients regarding the risks and benefits of continuing treatment and offer evidence‐based care when appropriate. Several studies highlight the significance of these discussions, as they have been found to correlate with less aggressive EOL care and higher quality of life for both patients and caregivers [9, 12, 19]. For instance, Wright et al. found that patients who participated in EOL discussions were 9% less likely to receive mechanical ventilation, 8% less likely to be admitted to the ICU, and 20% more likely to be enrolled in hospice for over 1 week [9]. This same study linked the receipt of aggressive EOL care to poorer patient quality of life, while their caregivers were at a three times higher risk of developing a major depressive disorder [9]. By failing to engage in GOC conversations and adequately document them, we are missing a crucial opportunity for patients to explore their treatment options, express their preferences for EOL care, and effectively communicate their needs to the broader care team.

There are several reasons why clinical trial subjects may be more likely to receive aggressive EOL care. In addition to infrequent documentation of GOC, factors such as decreased PC involvement likely contribute to these findings. Like GOC discussions, specialist PC involvement has also been shown to increase high‐quality EOL care and improve patient and caregiver quality of life [8, 10, 22]. Our study indicates that PC services continue to be underutilized (only 27.7% of patients referred) despite recommendations from organizations like ASCO, the National Comprehensive Cancer Network, and the Society for Gynecologic Oncology [23, 24, 25]. Patients enrolling in trials are even less likely to have the support of PC, with rates 6.3% less than their nontrial counterparts, which raises the question of whether a referral to PC should be considered a standard component of the trial enrollment process.

Overall hospice referrals were low across the study population, with less than one‐quarter of patients being referred before death. Notably, no significant differences were found in rates of hospice referrals, referrals within the last 3 DOL, or length of stay on hospice between cohorts. It is well‐documented that hospice enrollment is associated with improved quality of life, reduced symptom burden, and decreased instances of aggressive EOL care [26]. Additionally, prior studies have found that hospice enrollment of at least 90 days duration confers the maximum quality‐of‐life benefits to patients and families [27, 28]. In our study, patients enrolled in trials have a median time from hospice referral to death of 19 days. Although this is slightly higher than the national average length of stay on hospice for patients with cancer (18 days), there is vast room for improvement to ensure that patients and their families receive the maximum benefits from hospice care [26, 29].

Clinicians may argue that the identification of patients at the highest risk for death poses a challenge to initiating GOC conversations, referring patients to PC, and considering the use of hospice services. However, ample data show that simple tools like the Surprise Question (“Would I be surprised if this patient were to die in the next 12 months?”) can be utilized to effectively identify this high‐risk population across a variety of clinical and cancer settings [30, 31, 32, 33, 34]. In addition, ASCO recommends involving PC for patients diagnosed with advanced or recurrent cancers, and interventions to facilitate earlier referrals could help circumvent some of the prognostication challenges near the EOL [24].

Unexpectedly, our study suggests that patients enrolled in clinical trials toward the EOL were less likely to have an ED encounter in their last month of life. Patients enrolled in trials may have more frequent follow‐up with healthcare providers or trial coordinators, which may offer an added layer of support and an alternative means of triaging health concerns, avoiding ED encounters. These patients may also have higher healthcare literacy and thus be better equipped to know how and when to get help before things get bad enough to require an ED visit. Our suspicion, however, is that ED encounters for trial subjects may be underreported in our dataset, as we are only capturing data pertaining to one institution. Patients with advanced cancer may be more willing to travel further distances to enroll in a clinical trial [35]. Trial subjects have been shown to travel a median of 25 miles in each direction to enroll in a clinical trial, with 25% of National Institute of Health clinical trial patients traveling ≥ 100 miles each way to attend clinic visits [35]. We hypothesize that while these patients travel to our tertiary care center for their oncologic care, they may utilize community EDs closer to their homes for urgent concerns. As we have not collected data outside of our hospital system, we may be disproportionately undercounting ED encounters for those in our clinical trial cohort. Additionally, our data suggest that patients enrolled in clinical trials are less likely to die in an acute care setting. By similar logic, we feel this variable may also be underreported in our clinical trial cohort.

This study's strengths lie in its use of clinical and administrative data to analyze a comprehensive set of EOL care variables from a large patient population; however, its results must be considered within the context of its limitations. As this was a retrospective review of EHR data from a single tertiary care center, our findings are limited to the care received at our institution and may not be generalizable. Patients enrolling in clinical trials may be healthier at baseline to meet performance standards for trial enrollment [36, 37, 38]. Data pertaining to care received at outside institutions, such as ED encounters, were not captured in this study. Furthermore, GOC discussions documented outside of an advanced care planning note were not accounted for in this study.

Despite these limitations, we found that patients enrolled in clinical trials toward the EOL received more aggressive care, with higher rates of anticancer treatment toward the EOL, lower rates of PC engagement, and less frequent documentation of goals and care preferences. Our study highlights the need for continued quality improvement of EOL care, particularly around the utilization of hospice services, early involvement of specialty palliative care, and advancing training and empowerment of a variety of healthcare professionals to engage in longitudinal GOC discussions. Future research should focus on identifying and evaluating interventions to minimize aggressive EOL care and promote early integration of PC services and documentation of patient GOC, while focusing on the unique needs of various populations (particularly those with hematologic malignancies). The process of clinical trial enrollment could also include consideration of GOC discussions and referral to specialist PC as requirements for participation, particularly for patients with advanced or recurrent disease. Further analysis of EOL quality metrics and clinical trial phase may also identify populations at risk for goal‐discordant care. Additionally, future work should further characterize the impact of treatment‐related time toxicity on patient quality of life, especially for those enrolled in clinical trials [21].

## 5 Conclusion

Our study underscores the importance of aligning EOL care with patients' goals and preferences, particularly among those enrolling in clinical trials near the EOL. Despite established guidelines, our findings reveal increased initiation of anticancer treatments near the EOL and limited PC engagement and GOC documentation for those enrolled in clinical trials in their last year of life. Addressing these gaps through patient‐centered approaches to cancer care and early integration of PC services is essential to enhance the quality of EOL care for all patients.

## Author Contributions

**Melissa R. Rosen:** conceptualization (equal), data curation (lead), formal analysis (equal), investigation (equal), methodology (equal), project administration (supporting), writing – original draft (lead), writing – review and editing (equal). **Tracy Truong:** conceptualization (equal), investigation (equal), methodology (lead), validation (lead), visualization (lead), writing – review and editing (equal). **Catherine Gervais:** validation (equal), visualization (equal), writing – review and editing (supporting). **Thomas W. LeBlanc:** conceptualization (supporting), methodology (supporting), project administration (supporting), writing – review and editing (supporting). **Laura J. Havrilesky:** conceptualization (supporting), methodology (supporting), project administration (supporting), writing – review and editing (supporting). **Brittany A. Davidson:** conceptualization (equal), data curation (equal), formal analysis (equal), funding acquisition (equal), investigation (equal), methodology (equal), project administration (equal), supervision (equal), writing – original draft (equal), writing – review and editing (lead).

## Ethics Statement

All study activities occurred following Duke University Institutional Review Board approval (Pro00110974).

## Consent

Informed consent was granted an exemption by the institutional IRB for the purposes of this study.

## Conflicts of Interest

The authors declare no conflicts of interest.

## Data Availability

The data that support the findings of this study are available on request from the corresponding author. The data are not publicly available due to privacy or ethical restrictions.
